# Functional and quantitative evaluation of the 20S proteasome in serum and intracellular in145 moroccan patients with hematologic malignancies

**DOI:** 10.1186/s12014-022-09375-9

**Published:** 2022-11-15

**Authors:** Hassan Filali, Ouadie Mohamed El Yaagoubi, Ayoub Lahmadi, Asmaa Quessar, Said El Antri, Hamid Samaki, Souad Aboudkhil

**Affiliations:** 1 Laboratory of Biochemistry, Environment and Agrifood (URAC 36), Faculty of Science and Technology, Mohammedia-University Hassan II of Casablanca, Mohammedia, Morocco; 2 Hematology and Pediatric Oncology Service-Hospital August 20, UniversityHospital Center IBN ROCHD Casablanca, Casablanca, Morocco; 3 National Institute of Social Actions (INAS), Tanger, Morocco

**Keywords:** Proteasome, Hematologic malignancies, ELISA Assay, Chymotrypsin-likeactivity

## Abstract

**Background:**

Regulatory degradation of intracellular proteins plays an essential role in most biological processes, particularly in the control of cell proliferation and differentiation.

In eukaryotes, intracellular proteolysis is largely provided by the Ubiquitin / Proteasome system. Alterations and dysfunction of protein degradation by the Ubiquitin / Proteasome system, such as transcription factors, cell cycle regulators or tumor suppressor proteins, have been linked to human.

Pathologies, including blood cancers. Mainly localized in the nucleus and cytoplasm of cells, the proteasome can be detected in the cell culture supernatant or in the peripheral blood of patients.

This study deals with the problems of the search for serum markers specific to certain pathologies and which would be useful in the prevention, diagnosis and monitoring of cancers and which could be used as a therapeutic tool.

**Methods:**

The functional and quantitative analysis of the proteasome is carried out at the serum and subcellular level during a pathological phenomenon in a population of 145 Moroccan patients (sex ratio: 1.10 / average age: 47.9 ± 15, 3 years) using an indirect ELISA test and a follow-up of the fluorescence emitted after enzymatic digestion of specific peptides by proteolytic activity (chymotrypsin-like).

**Results:**

The evolutionary trend proteasome subcellular is significantly linked to the rate of chymotrypsin-like activity. The entire population of 60 patients called back for a second blood test.

After three months of treatment reported a significant drop in the rate and the activity of the proteasome in serum and intracellular level.

**Conclusions:**

Although the serum proteasome level is a potential new tool for the monitoring of.

Patientswithliquid cancer.

*Trial registration*: retrospectively registered.

## Introduction

Every cell is a complex system that maintains its homeostasis by constantly remodeling its proteome. It adapts to its environment by producing new proteins and renewing or eliminating obsolete or defective proteins [1]. Indeed, intracellular proteolysis is involved in the elimination of abnormal proteins, in the supply of amino acids required for the synthesis of novel proteins in the context of metabolic control, in the maturation of polypeptide precursors [2, 3], in the immune response through the production of peptides presented on the cell surface by the major histocompatibility complex class I, and also in proliferation and programmed cell death [4]. To date, lysosomes, calpain, caspases and the ubiquitin–proteasome system are the four main intracellular proteolytic systems [5].

In eukaryotes, intracellular proteolysis is largely provided by the ubiquitin/proteasome system (80%). This system includes hundreds of components that target proteins for degradation [6]. The proteasome is localized in the nucleus and cytoplasm of eukaryotic cells, although its subcellular distribution varies from one organ to another [7]. Because the ubiquitin–proteasome system interferes with various cellular metabolic processes, deregulation of these components can cause many diseases, including neurodegenerative diseases, certain cancers, and autoimmune diseases [8, 9].

Many research teams have focused on quantifying proteasome release in peripheral blood and have demonstrated high proteasome levels in patients with autoimmune diseases, proliferative lymphoid syndromes, solid tumors [10] or renal carcinomas compared to controls [11]. Although the bibliographic data strongly confirm the relationship between the proteasome and certain cancers, including the use of proteasome inhibitors for the control of certain forms of tumors [12], already satisfaction (multiple myeloma).In others, hematologic malignancies, the data are still controversial.

We planned this longitudinal study in a large cohort of the Moroccan population suffering from different forms of neoplastic diseases before and after chemotherapy. The aim was to analyze the developmental tendency of the proteasome and its proteolytic activity (chymotrypsin-like activity) in the peripheral blood and at the intracellular level, thus supporting the data from the bibliography on the possibility of using this parameter as a biological indicator of the developmental status of liquid tumors.

## Material and method

### Patients

The study included 145 Moroccan patients (sex-ratio: 1.10;Average age: 47.9 ± 15.3 years) with various forms of hematological malignancies, all newly diagnosis and untreated (Table 1). Blood samples were collected, after obtaining informed consent from all recruited patients between April 2012 and June 2014 at the Department of Hematology and Oncology Pediatric—Hospital 20 August Casablanca –Hospital Centre University IBN ROCHD Casablanca.

**Table 1:** Clinical characteristics of the study population

Diagnostic	N	Average age	Male	Female	Untreated	Treatment
ML						
AML	45	40,6 ± 16,5	24	21	32	13
CML	10	45,4 ± 13,3	5	5	6	4
LL						
ALL	14	31,4 ± 14,8	8	6	6	8
CLL	9	58,4 ± 16,2	6	3	4	5
MM	18	60,3 ± 10,3	9	9	10	8
NHL	28	51,8 ± 18,3	12	16	16	12
HD	21	48 ± 18,2	12	9	11	10
Total	145	47,9 ± 15,3	76	69	85	60

*ML* myeloidlineage, *AML* acute myeloid leukemia, *CML* chronic myeloid leukemia, *LL* lymphoidlineage, *ALL* acute lymphoid leukemia, *CLL* chronic lymphocytic leukemia, *MM* multiple myeloma, *NHL* non-Hodgkin's lymphoma, *HD* Hodgkin's disease

A population of 60 patients with different forms of Hematologic Diseases was recalled for a second blood test after three months of treatment.

Patients with leukemia are divided into two classes: acute leukemia (AL) and chronic leukemia (CL). Hodgkin lymphoma patients and non-Hodgkin were grouped into a single category: Lymphoma.

### Preparation of samples

The Serum is obtained from a fresh sample of blood human and gradually cooled to 4 °C and − 20 °C. The lymphocyte layer is obtained by lysis of the erythrocytes or by Ficoll gradient.

### Lysis of the cells

Briefly we broke out the red blood cells from the blood sample, by adding a solution of Tris/ EDTA (20/5) after centrifugation**,** we eliminate the supernatant and repeat this maneuver until obtaining a clear cell layer.

Added quickly 200 µl of Lysis buffer (10 mMNaCl, 10 mMKcl, HEPES 10 mM, EDTA 1 mM PH 7.1, DTT 0.1 mM, 1% Triton, fortified with Anti proteases (PMSF 2 nM) on each sample. Cells are exploded with periods of 20 s break alternated with 30 s rest to avoid the excessive heating of the samples that may cause a denaturation of enzymes. The Protocol is repeated 3 times. The extracts can be stored at − 20 °C until their use [12].

### Quantification of the proteasome 20 s human by ELISA

Determination of the serum and intracellular, 20 s Proteasome by the technique of Indirect ELISA [13] based on recognition in “sandwich” of complexes of 20 s proteasome by specific antibodies of the subunit α of the catalytic core: MCP20 (Enzolifesciences).First, there is the ‘Coating’ of the monoclonal Ab MCP20 (1/3000) to the microplate 1 h at 37 °C. Non-specific sites are blocked by addition of 200 µl of PBS—BSA 2%. Are deposited the standard range and the samples to be determined (100 µl / well). Subunits of the proteasome captured are detected by second polyclonal Ab I57I (1: 5000). The revelation is made after adding conjugate labeled with peroxidase. After the addition of the substrate (OPD), is the follow-up to the OD at 492 nm on an ELISA reader (ELx800 UV). The results are expressed in concentration of Proteasomes in: ng/ml, after comparison with the standard curve obtained with the standard of the proteasome 20 s purified human range.

### Catalytic activity of the proteasome (chymotripsyne-like)

Catalytic activity (chymotrypsin-like) of the 20 s proteasome, is determined by measuring substrate fluorogenic (AMC) released after degradation of the peptideSuccinyl-leu-leu-val-tyr-amido-4-methyl-Coumarin “Suc-LLVY-AMC” Sigma Aldrich), after 3 h of incubation of the substrate (40 µM) in Proteasome assay Buffer (25 mM HEPES (pH 7.5), 0.5 mM EDTA), with the same volume of the sample containing the proteasome (serum or cell lysate) at 37 °C (12).

The fluorescence emitted after cleavage of the AMC is measured on a Fluorometer (Heofer Scientific Instruments). The excitement of peptides, coupled at the AMC, is done through a 360/40 nm filter that the wavelength is between 340 and 380 nm with a maximum at 360 nm. The fluorescence emitted after digestion of peptides coupled to the AMC reads on the 460/40 nm filter.

To convert the unit of fluorescence (UF) issued μmol of used fluorophores, a standard AMC range established from a stock solution of AMC at 10^−3^ M. The excitement of the AMC is done at a wavelength of 360/40 nm and the signal is then recovered on the 460/40 nm filters.

### Other biological parameters analyzed

In Patients with hematological malignancies, three biological parameters were tested and analyzed, in order to establish possible statistical correlation with the level of circulating and intracellular 20 s proteasome: Lactate Dehydrogenase (LDH) as global marker of cytolysis.

### Statistics

Comparison of proteasome 20 s serum and intracellular concentrations, all data obtained are represented as the mean ± Standard Error of the Mean (SEM). Student’s *t*-test was used to test whether differences between two groups were significant.

## Results

### I-Simultaneous evolution of the proteasome in serum according to the chymotrypsin-like activity

**Fig. 1:**
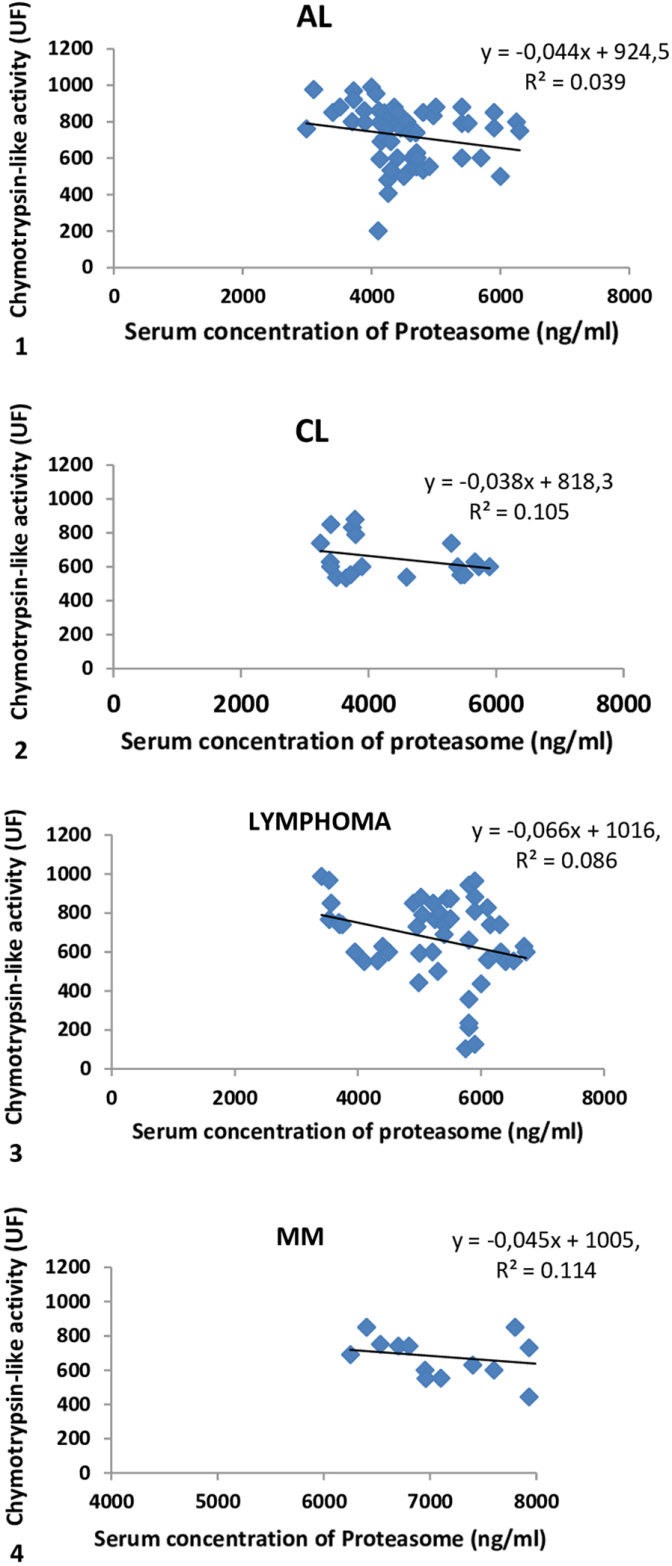
Correlation between serum concentration of proteasome and the chymotrypsin-like activity: in patients with AL^1^ and CL^2^, LYMPHOMA^3^, andMM^4^

Serum Proteasome measured by ELISA, in 145 patients (with an average age 47.9 ± 15.3 years) suffering from Multiple Myeloma,Lymphoma, Acute Leukemya and Chronic Leukemia according to the chymotrypsin-like activity is represented in Fig. 1.

All patients with Hematologic malignancies express proteolysis rate more pronounced in serum compared to the control.

However, analysis of the correlation between chymotrypsin-like activity and the concentration of the serum proteasome shows no logical link with all types of Hematologic Diseases.

### II-Simultaneous evolution of the subcellular proteasome according to the chymotrypsin-like activity

**Fig. 2:**
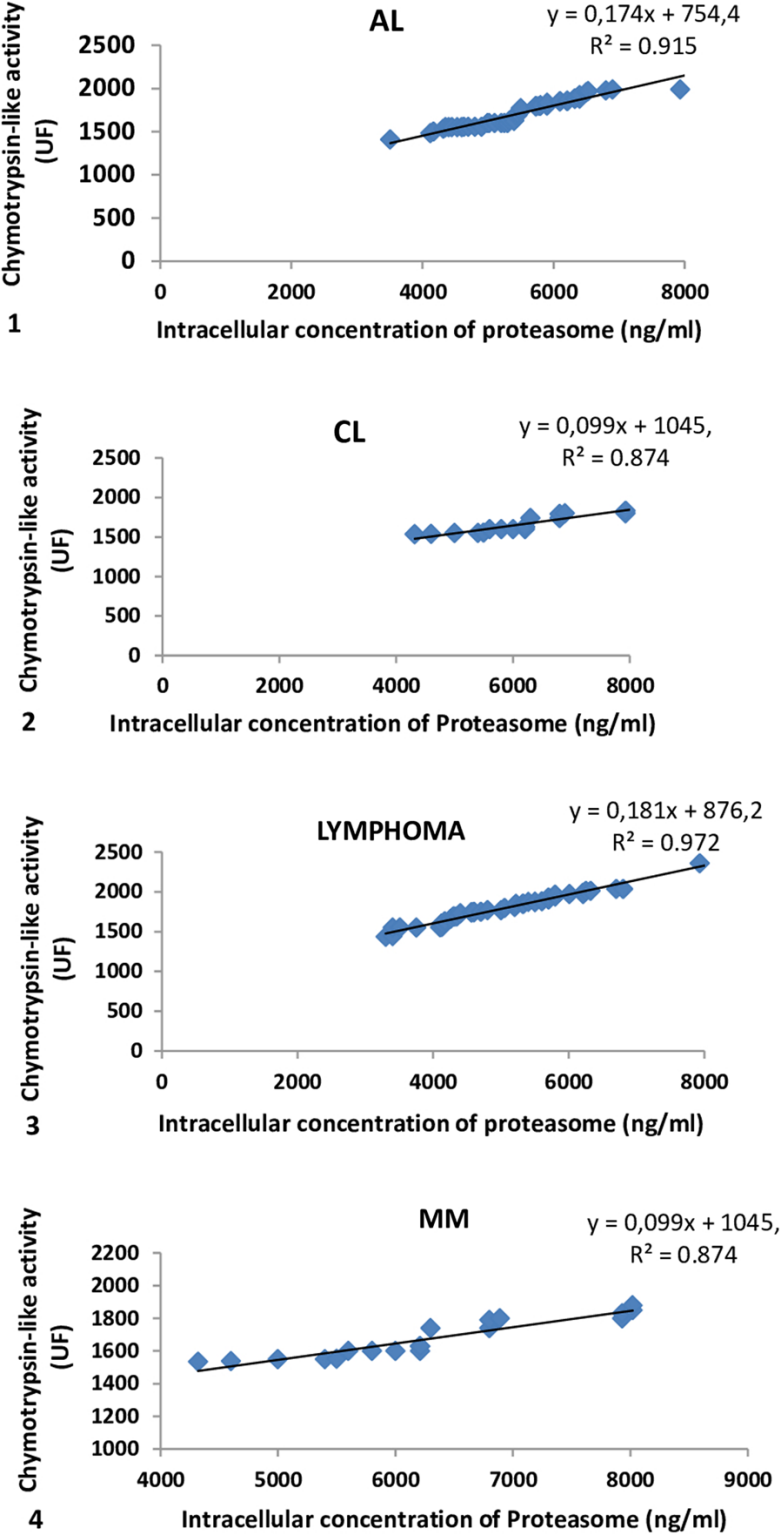
Correlation between intracellular concentration of proteasome and the chymotrypsin-like activity: in patients with AL^1^ and CL^2^, LYMPHOMA^3^, andMM^4^

The rate of intracellular Proteasome, in patients, reached to hematological malignancies according to the chymotrypsin-like activity is represented in Fig. 2

Furthermore, the analysis of changes in intracellular chymotrypsin-like activity based on intracellular proteasome (ng/ml), reveals a positive evolutionary trend and those in all recruited patients (CL, AL, MM and Lymphoma).

### III-Influence of treatment on the evolution of the proteasome at the Hematological malignancies

#### Proteasome concentration and chymotrypsine-like activity in serum

The proteasome, a key element of the neoplastic differentiation, currently attracting great interest as a new marker in different pathologies.

A population of 60 patients with different forms of Hematologic Diseases was recalled for a second blood test after three months of treatment.

**Fig. 3:**
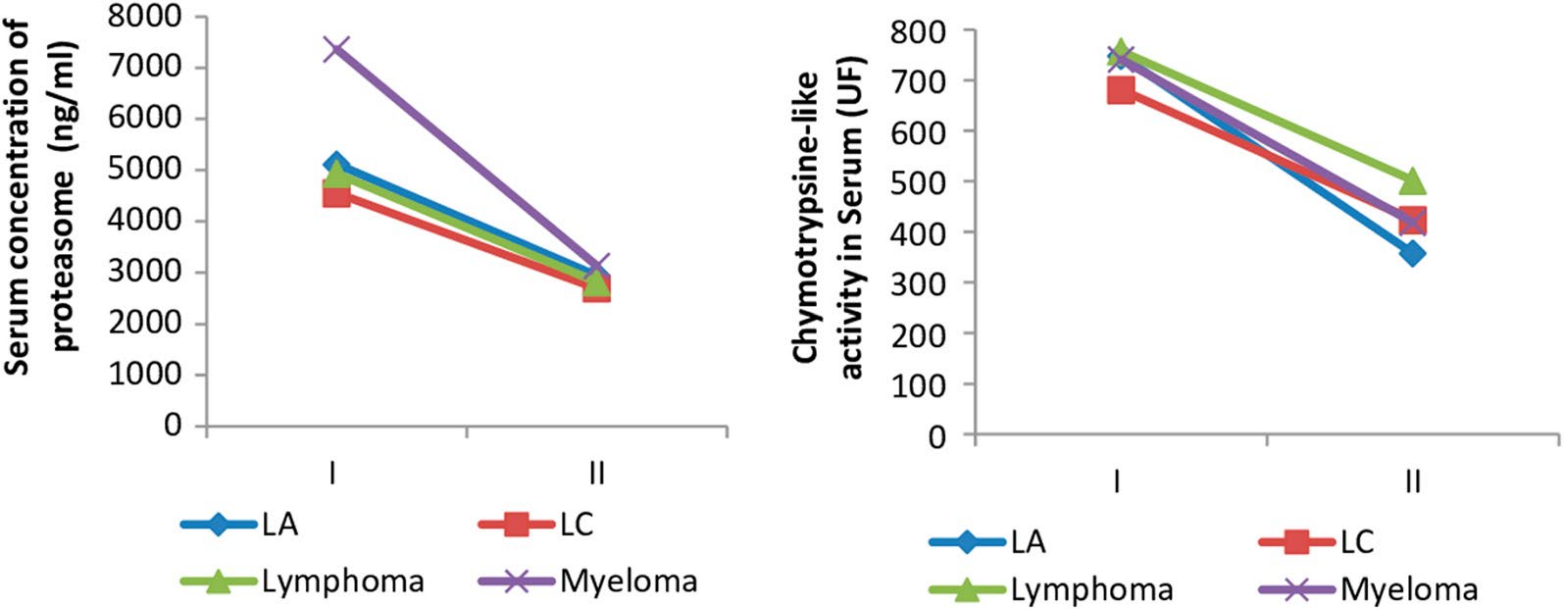
Change in serum concentration of proteasome and proteolytic activity depending on the type of pathology (I: before treatment; II: under treatment)

After 3 months of treatment, all patients with Hematological malignancies, express a serum proteasome levels significantly low compared to stage I (no treatment).The patients with MM and AL show a very significant regression respectively 57% and 45%. A reduction in serum catalytic activity was observed in all patients who have undergone treatment. A sharp drop in catalytic activity (60%) was observed in patients with AL. The patients with lymphoma, MM, CL, display a respective decrease of 47%, 37%, 35% (Fig. 3).

#### Subcellular rate of proteasome and chymotrypsine-like activity

All Subjects with Hematologic malignancies, having received an anti-tumor treatment, recalled after a three-month period, have levels of intracellular proteasome significantly reduced compared to the values of stage I (untreated).

**Fig. 4:**
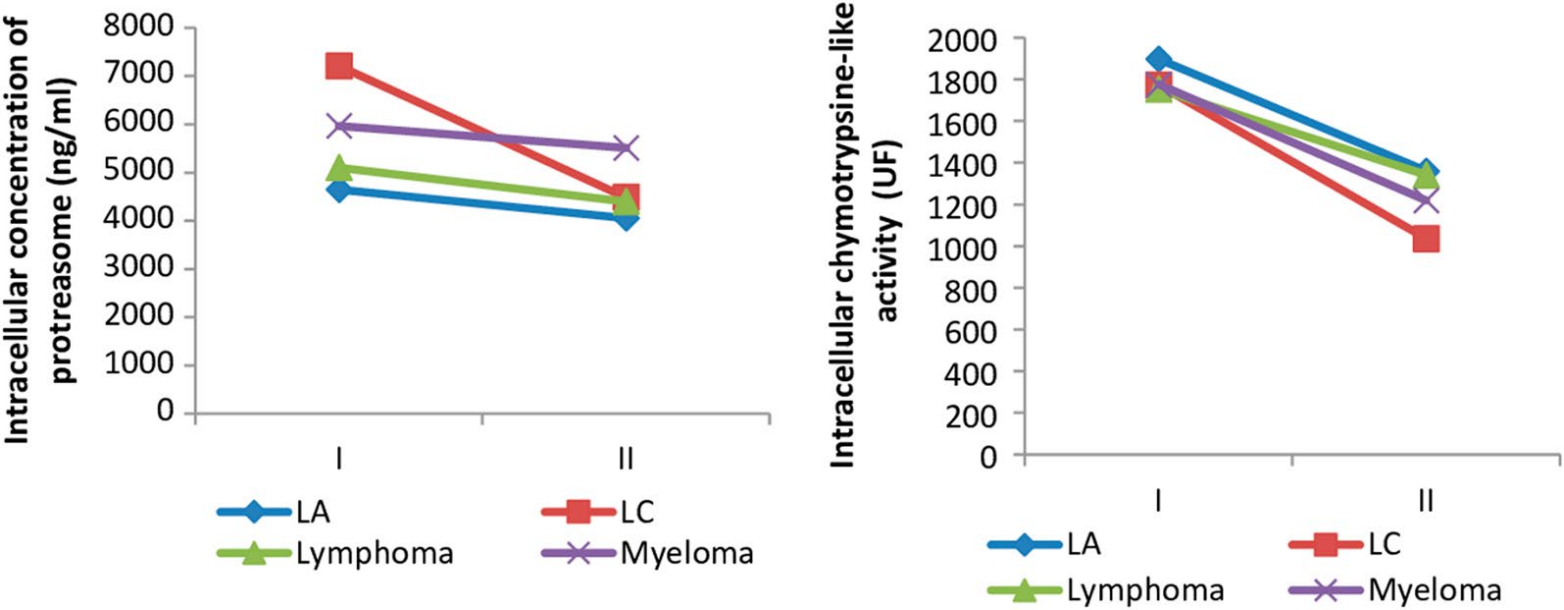
Change in sub cellular concentration of proteasome and proteolytic activity depending on the type of pathology (I: before treatment; II: under treatment)

In fact there was a decrease in intracellular concentration of the most important proteasome in patients with CL, about 41%, followed by lymphoma patients display decreased proteasome rate of 29%, followed by the patients with AL showing a decrease of 16.5%; and patients with myeloma, which has the lowest rate reduced proteasome 10%

we find that patients with CL, shows a highest percentage decrease in catalytic activity (about 46%) after taking cancer treatment, followed those with Myeloma display a decrease in the catalytic activity of about 40%, patients with AL with a reduction rate of 31% and lymphoma patients with a decrease of 25% of the catalytic activity.(Fig. 4).

#### Quantitative and functional evolution of the proteasome depending on the origin of tumor cells

We subdivided our cancer patient population into two categories based on the origin of the tumor cell line: myeloid lineage (n = 54) included patients with AML and CML and Lymphoid line (n = 92) Lymphoma, Myeloma and CLL.A significant decrease in serum levels of the proteasome was observed in the Myeloid lineage (concentration: 4116 ± 702 ng/ml) compared to the Lymphoid lineage (concentration: 5554 ± 512 ng/ml; p < 0.05).

In addition, there was a significant difference (P < 0.001) in the mean intracellular proteolytic activity between the Lymphoid and Myeloid line.

**Fig. 5:**
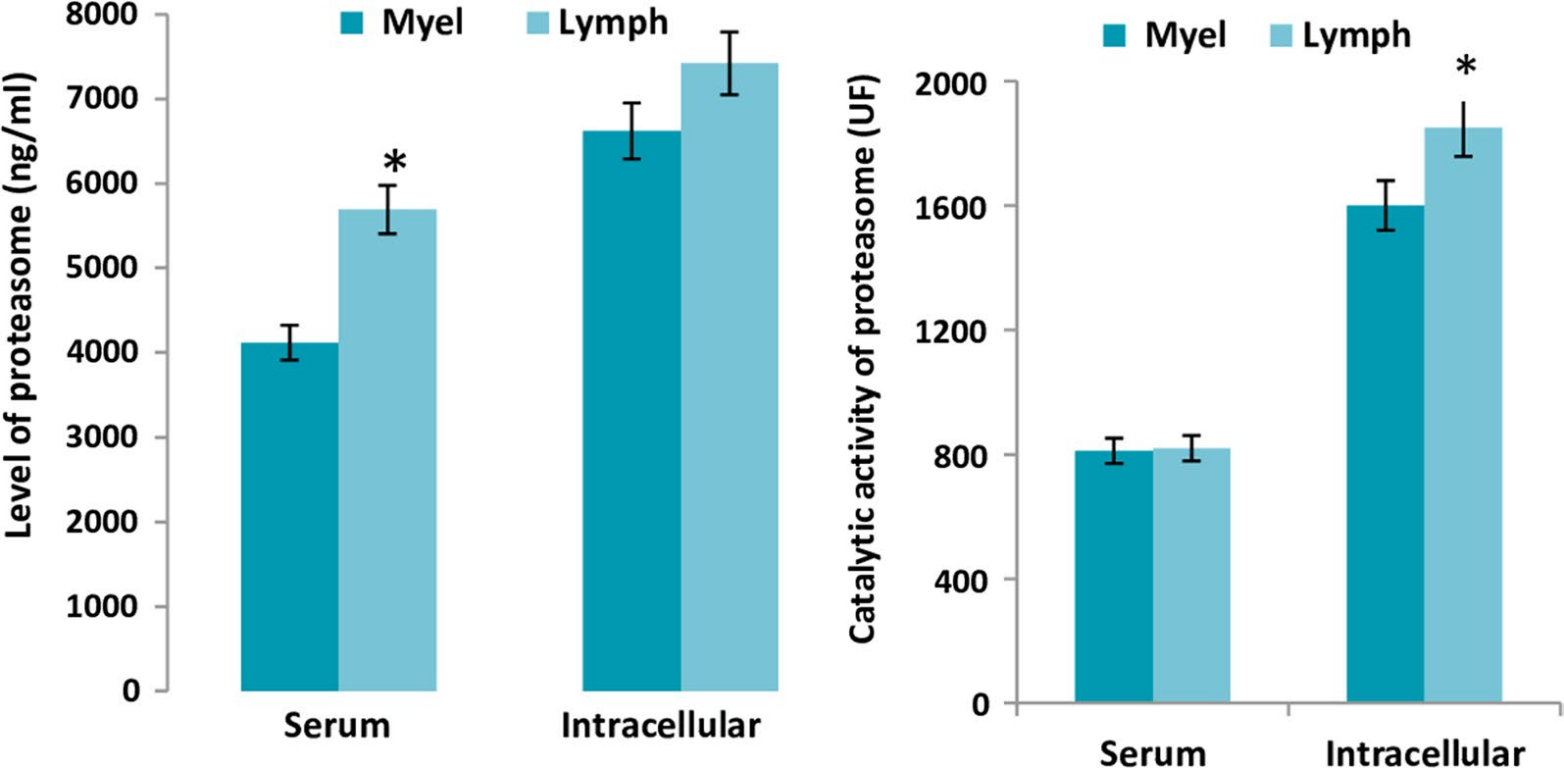
Comparison of the rate and catalytic activity of proteasome between Myeloid and Lymphoid Lineage at sub cellular and serum level; *: P < 0.05 compared to the myeloid lineage

No significant difference (p > 0.05) was observed between the Lymphoid and Myeloid line in the subcellular proteasome concentration or serum proteolytic activity (Fig. 5).

#### Quantitative and functional evolution of the proteasome depending on the evolutionary stage of the disease in lymphomaafter-treatment

We classified our patient population suffering from lymphoma (n = 22), depending on the stage of tumor progression (according to Ann Arbor classification) intotwo types (1: Class I and II, and 2: Class III).

**Fig. 6:**
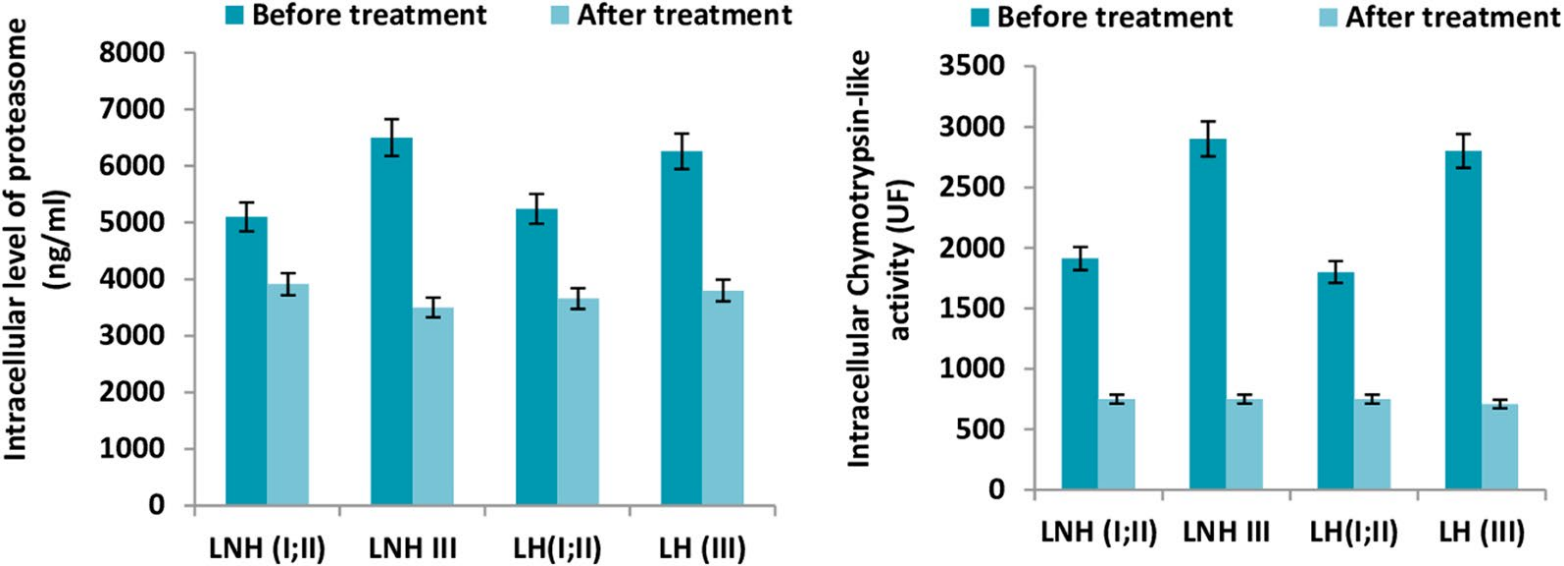
Comparison of the rate and catalytic activity of proteasome between LNH and LH in sub cellular level

After that we analyzed the quantitative and functional evolutionary trend of the proteasome after taking the treatment, we noted that patients suffering from lymphoma class III report a significant regression (49%) after treatment (before: 6450 ± 940 ng/ml; after 3600 ± 560 ng/ml in the patients with NHL;before 6500 ± 760 ng/ml; after 3850 ± 550 ng/ml in patients with HL).Otherwise a regression(27%) is recorded in stage I and II lymphoma (NHL and HL). The same situation is observed at the chymotrypsin-like activity (Fig. 6).

#### Functional evolution of proteasome according to a biological parameters (LDH)

In patients with hematological malignancies, a biological parameters was tested and analyzed, in order to establish possible statistical correlation with the level of circulating and intracellular 20 s proteasome: Lactate Dehydrogenase (LDH) as global marker of cytolysis.

**Fig. 7:**
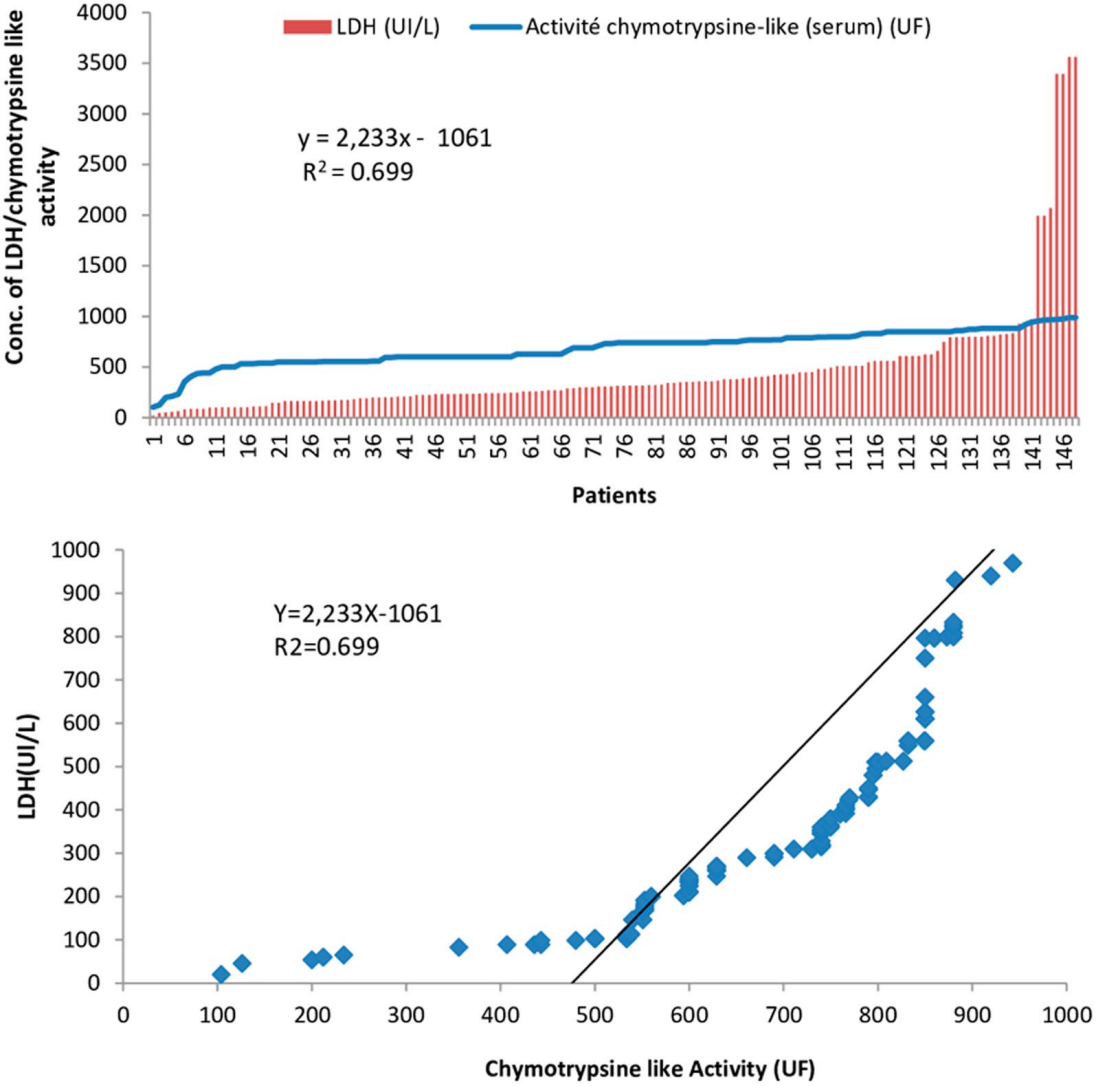
Simultaneous Evolution of serum chymotrypsin-like activity (UF) and the rate of LDH (UI/ l) in patients with Hematologic malignancies

A progressive evolutionary trend is observed between the activity of the proteasome and LDH released into circulation (Fig. 7). Otherwise the linear correlation report significant dependence average (R^2^ = 0.699).

## Discussion

Recently it has been shown that protein degradation by the proteasome is a biological process that is fundamental to the survival ofcancer cells [11]. The proteasome activity contained in a lysate can be modulated by different proteins in the cell, whereas theactivity measured on the purified 20S proteasome reflects only the specific activities of this complex [14].

The relationship between clinical condition and concentration of the proteasome has been reported in several diseases, particularly in patients with systemic lupus erythematosus, rheumatoid arthritis [15] and also in the case of solid tumors and blood cancers [16, 17]. In 2004, Adams reported that the rapid and large storage misfolded proteins and/or mutant is certainly the origin of the dependence of malignant cells onan active intracellular proteasome [7]. Indeed, Matondo in 2010 showed that the most immature cells (KG1a) possess a greater amount of proteasome than the more mature cells (U937). Whereas the chymotrypsin-like activity of the proteasome increases with the degree of cell maturation [14].

The catalytic activity is the main function of the proteasome, and its analysis serves both to learn about the intracellular and extracellular state of the proteasome and to respond to the effectiveness of treatment [18]. Furthermore, the analysis of changes in the proteasome activity level in serum and intracellular in all patients shows a gradual developmental trend. Furthermore the intense proteasome activity observed in tumor cells seems to be particularly involved in apoptosis resistance, which is a common feature of most malignant tumors [19, 20]

Treatment with proteasome inhibitors remains financially very expensive and inaccessible for the vast majority of Moroccan patients, making it very difficult to assemble a valid sample to analyze the direct effects of treatment with a proteasome inhibitor (Bortezomib). Proteasome development was analyzed in patients after three months of treatment, abstracting for the type of treatment.

All treated patients showed a significant regression of the serum proteasome level. In fact, the values obtained within the population of MM patients are similar to those published by Jakob et al. 2007 [17, 21] concluding that the level of circulating proteasome is correlated at the stage of the disease and that the effectiveness of the chemotherapy.

The reduction in serum proteasome activity and levels after treatment mainly affects patients with MM, LA. At the subcellular level, patients with CL, lymphoma and LA reported the greatest reduction. In patients with acute leukemia, NHL and MM, decrease in serum was much more pronounced in the serum than at the subcellular level.

Actually, it’s well established that treatment with inhibitors of proteolytic activity of the proteasome leads to positive results in the treatment of certain liquid tumors particularly myeloma [17]. Indeed, a significant decrease in circulating proteasome levels (> 60%) was observed in all patients who received chemotherapy and in patients in full remission blood cancer compared to controls [10].

The research initiated by the team (Lavabre-Bertrand et *al*. Which reports the direct correlationbetween the level of circulating proteasome and tumor cell lysis in neoplastic diseases, strongly supports the positive correlation observed between LDH level and proteasome activity of the proteasome at the serum level in our study population, as it is well established that myelo-proliferative disorders involve a high rate of intramedullary apoptosis [22, 23].

The proteasome level correlates with the clinical status of the patient and the stage of development of the hematologic [24, 25] The decrease in serum proteasome level after treatment may be related to the process of massive relocation of proteasome subunits into cytoplasmic storage granules proteasome [26]. This rapidly reverses upon re-entry into cell proliferation [27]. Cancer is the result of aberrant cell accumulation fitness [28, 29]

The use of the circulating proteasome assay as a tumor biomarker is a tool that could be very satisfactory for monitoring patients after remission in order to prevent a potential risk of falling. However, it remains problematic because absolute levels of the proteasome vary widely from one laboratory to another. These observed changes may be technical because they may be related to the nature of the serum proteasome [29, 30].

## Conclusion

Changes in proteasome activity and their possible involvement in the development of diseases have been proved in many research recently published especially in Blood cancer. The use of proteasome circulating assay as a biomarker of tumor and astool that could be very satisfying to follow patients after remission and prevent possible fall. The catalytic activity is the main function of the proteasome, its analysis allows to inform aboutthe state of the intracellular- and extracellular proteasome and to respond to the efficacy of a treatment.

## Data Availability

Not applicable.

## References

[1] Saez I, Vilchez D (2014). The mechanistic links between proteasome activity aging and age-related diseases. Curr Genomics.

[2] El Yaagoubi OM, Oularbi L, Bouyahya A, Samaki H, El Antri S, Aboudkhil S (2021). The role of the ubiquitin-proteasome pathway in skin cancer development: 26S proteasome-activated NF-κB signal transduction. Cancer Biol Ther.

[3] Ito S (2021). Proteasome inhibitors for the treatment of multiple myeloma. Cancers.

[4] Tonoki A, Kuranaga E, Tomioka T, Hamazaki J, Murata S, Tanaka K (2009). Genetic evidence linking age-dependent attenuation of the 26s proteasome with the aging process. Mol Cell Biol.

[5] El Yaagoubi OM, Lahmadi A, Bouyahya A, Filali H, Samaki H, El Antri S, et al. Antitumor effect of inula viscosa extracts on dmba-induced skin carcinoma are mediated by proteasome inhibition. BioMed Research International 2021 10.1155/2021/6687589 PMC8019636 33855081

[6] Chowdhury M, Enenkel CI (2015). ntracellular dynamics of the ubiquitin-proteasome-system. F1000Res.

[7] Adams J (2004). The proteasome: a suitable antineoplastic target. Nat Rev Cancer.

[8] Sultana S, Bouyahya A, Rebezov M, Shariati MA, Balahbib A, Khouchlaa A (2022). Impacts of nutritive and bioactive compounds on cancer development and therapy. Crit Rev Food Sci Nutr.

[9] Enenkel C (1843). Proteasome dynamics. Biochim Biophys Acta.

[10] Lavabre-Bertrand T, Henry L, Carillo S, Guiraud I, Ouali A, Dutaud D (2001). Plasma proteasome level is a potential marker in patients with solid tumors and hemopoietic malignancies. Cancer.

[11] de Martino M, Hoetzenecker K, Ankersmit HJ, Roth GA, Haitel A, Waldert M (2012). Serum 20S proteasome is elevated in patients with renal cell carcinoma and associated with poor prognosis. Br J Cancer.

[12] Matondo M, Bousquet-Dubouch M-P, Gallay N, Uttenweiler-Joseph S, Recher C, Payrastre B, et al. Proteasome inhibitor-induced apoptosis in acute myeloid leukemia: a correlation with the proteasome status. 10.1016/j.leukres.2009.09.020 19811823

[13] Dutaud D, Aubry L, Henry L, Levieux D, Hendil KB, Kuehn L (2002). Development and evaluation of a sandwich ELISA for quantification of the 20S proteasome in human plasma. J Immunol Method.

[14] Magill L, Lynas J, Morris TC, Walker B, Irvine AE (2004). Proteasome proteolytic activity in hematopoietic cells from patients with chronic myeloid leukemia and multiple myeloma. Haematologica.

[15] Egerer K, Kuckelkorn U, Rudolph PE, Rückert JC, Dörner T, Burmester G-R (2002). Circulating proteasomes are markers of cell damage and immunologic activity in autoimmune diseases. J Rheumatol.

[16] Henry L, Lavabre-Bertrand T, Vercambre L, Ramos J, Carillo S, Guiraud I (2009). Plasma proteasome level is a reliable early marker of malignant transformation of liver cirrhosis. Gut.

[17] Jakob C, Egerer K, Liebisch P, Turkmen S, Zavrski I, Kuckelkorn U (2007). Circulating proteasome levels are an independent prognostic factor for survival in multiple myeloma. Blood.

[18] Berkers CR, Verdoes M, Lichtman E, Fiebiger E, Kessler BM, Anderson KC (2005). Activity probe for in vivo profiling of the specificity of proteasome inhibitor bortezomib. Nat Methods.

[19] Spano J-P, Bay J-O, Blay J-Y, Rixe O. Proteasome inhibition: a new approach for the treatment of malignancies. Bulletin du Cancer. 16316823

[20] Choi WH, Kim S, Park S, Lee MJ. Concept and application of circulating proteasomes. Exp Mol Med. Nature Publishing Group 2021 10.1038/s12276-021-00692-x PMC8568939 34707192

[21] Maruyama H, Hirayama K, Yamashita M, Ohgi K, Tsujimoto R, Takayasu M (2020). Serum 20S proteasome levels are associated with disease activity in MPO-ANCA-associated microscopic polyangiitis. BMC Rheumatol.

[22] Shah S, Mudireddy M, Hanson CA, Ketterling RP, Gangat N, Pardanani A (2017). Marked elevation of serum lactate dehydrogenase in primary myelofibrosis: clinical and prognostic correlates. Blood Cancer J.

[23] Lavabre-Bertrand T, Henry L, Guiraud I, Carillo S, Bureau JP (2000). The proteasome and malignant hemopathies. Morphol Bullet Assoc Anatom.

[24] Sixt SU, Dahlmann B (2008). Extracellular, circulating proteasomes and ubiquitin - incidence and relevance. Biochim Biophys Acta.

[25] McBride A, Ryan PY (2013). Proteasome inhibitors in the treatment of multiple myeloma. Expert Rev Anticancer Ther.

[26] Chavrier P, Mamessier É, Aulas A. Les granules de stress, des acteurs émergents en cancérologie. médecine/sciences [Internet]. EDP Sciences; 2021 10.1051/medsci/2021109 34491181

[27] Laporte D, Salin B, Daignan-Fornier B, Sagot I (2008). Reversible cytoplasmic localization of the proteasome in quiescent yeast cells. J Cell Biol.

[28] Le Gall J-Y, Ardaillou R (2009). Biologie du vieillissement. Bulletin Acad Nation Méd.

[29] Almond JB, Cohen GM (2002). The proteasome: a novel target for cancer chemotherapy. Leukemia.

[30] Janse van Rensburg HJ, Spiliopoulou P, Siu LL (2022). Circulating biomarkers for therapeutic monitoring of anti-cancer agents. Oncologist.

